# Predicting sepsis in-hospital mortality with machine learning: a multi-center study using clinical and inflammatory biomarkers

**DOI:** 10.1186/s40001-024-01756-0

**Published:** 2024-03-06

**Authors:** Guyu Zhang, Fei Shao, Wei Yuan, Junyuan Wu, Xuan Qi, Jie Gao, Rui Shao, Ziren Tang, Tao Wang

**Affiliations:** Emergency Medicine Clinical Research Center, Beijing Chaoyang Hospital, Capital Medical University, Beijing Key Laboratory of Cardiopulmonary Cerebral Resuscitation, Beijing, 100020 China

**Keywords:** Sepsis, Prediction, Machining learning, Intensive care unit, XGBoost

## Abstract

**Background:**

This study aimed to develop and validate an interpretable machine-learning model that utilizes clinical features and inflammatory biomarkers to predict the risk of in-hospital mortality in critically ill patients suffering from sepsis.

**Methods:**

We enrolled all patients diagnosed with sepsis in the Medical Information Mart for Intensive Care IV (MIMIC-IV, v.2.0), eICU Collaborative Research Care (eICU-CRD 2.0), and the Amsterdam University Medical Centers databases (AmsterdamUMCdb 1.0.2). LASSO regression was employed for feature selection. Seven machine-learning methods were applied to develop prognostic models. The optimal model was chosen based on its accuracy, F1 score and area under curve (AUC) in the validation cohort. Moreover, we utilized the SHapley Additive exPlanations (SHAP) method to elucidate the effects of the features attributed to the model and analyze how individual features affect the model’s output. Finally, Spearman correlation analysis examined the associations among continuous predictor variables. Restricted cubic splines (RCS) explored potential non-linear relationships between continuous risk factors and in-hospital mortality.

**Results:**

3535 patients with sepsis were eligible for participation in this study. The median age of the participants was 66 years (IQR, 55–77 years), and 56% were male. After selection, 12 of the 45 clinical parameters collected on the first day after ICU admission remained associated with prognosis and were used to develop machine-learning models. Among seven constructed models, the eXtreme Gradient Boosting (XGBoost) model achieved the best performance, with an AUC of 0.94 and an F1 score of 0.937 in the validation cohort. Feature importance analysis revealed that Age, AST, invasive ventilation treatment, and serum urea nitrogen (BUN) were the top four features of the XGBoost model with the most significant impact. Inflammatory biomarkers may have prognostic value. Furthermore, SHAP force analysis illustrated how the constructed model visualized the prediction of the model.

**Conclusions:**

This study demonstrated the potential of machine-learning approaches for early prediction of outcomes in patients with sepsis. The SHAP method could improve the interoperability of machine-learning models and help clinicians better understand the reasoning behind the outcome.

**Supplementary Information:**

The online version contains supplementary material available at 10.1186/s40001-024-01756-0.

## Introduction

Sepsis is a severe illness that arises from various infections, leading to uncontrolled systemic inflammation. Despite medical advances and increased knowledge of its pathophysiology, sepsis remains a common cause of ICU admission and causes over 30 million deaths annually [1, 2]. According to the third international consensus definition, sepsis and septic shock are rapidly progressive inflammatory conditions accompanied by a state of immunosuppression [3]. Neutrophils are primary effector cells during systemic inflammatory reactions and exert regulatory roles over other immune cells by secreting cytokines and chemokines that enhance their recruitment, activation, and function [4, 5]. The neutrophil-to-lymphocyte ratio (NLR), calculated as a simple ratio between the neutrophil and lymphocyte counts measured in peripheral blood, reflects two aspects of the immune system: innate immunity, predominantly mediated by neutrophils, and adaptive immunity, supported by lymphocytes6. The NLR has acted as a reliable diagnostic marker for bacteremia and sepsis [7], with higher NLR values associated with adverse prognoses in patients with sepsis [8]. Moreover, NLR values have demonstrated a potential effect in assessing sepsis severity, notably elevated in patients with septic shock. Recent researchers have explored prediction models based on NLR, revealing their excellent diagnostic and prognostic capabilities in sepsis [9–11].

Compared with other markers, such as C-reactive protein (CRP) and white blood cell count (WBC), NLR exhibited moderate sensitivity and high specificity [12]. High-density lipoprotein (HDL), known for its anti-inflammatory properties, has been demonstrated to have prognostic implications in patients with inflammatory disorders, including sepsis [13]. HDL levels significantly decrease during sepsis, and low HDL correlates with higher hospital mortality, likely due to its anti-inflammatory properties [14]. Furthermore, recent studies have shown that immune-inflammation markers such as platelet-to-lymphocyte ratio (PLR), lymphocyte-to-monocyte ratio (LMR), NLR, monocyte/high-density lipoprotein cholesterol ratio (MHR) have garnered attention in identifying patients with septic and other infectious diseases [15–18]. Machine learning is a synthesis of mathematical methods that seek to distill knowledge and insights from large datasets to develop algorithms capable of predicting outcomes through data-driven ‘learning.’ This paradigm significantly outperforms traditional statistical methods regarding predictive accuracy [19]. For example, Ren and Yao et al. described how the XGBoost model outperformed a stepwise logistic regression model in predicting in-hospital mortality by identifying key features associated with outcomes such as coagulopathy, fluid electrolyte disturbances, renal replacement therapy (RRT), urine output, and cardiovascular surgery [20]. Similarly, the XGBoost model was touted as a reliable tool for predicting acute kidney injury (AKI) in septic patients, demonstrating superior performance over six other machine learning models [21]. In addition, John and Aron have developed a machine learning scoring method to predict the onset of sepsis within 48 h, a novel approach aimed at identifying at-risk populations, tailoring clinical interventions, and improving patient care [22]. Considering these findings, our study seeks to extend this emerging field by introducing features associated with immune-inflammation biomarkers. Incorporating these features may reveal new insights into the pathophysiology of sepsis and create more accurate prognostic models. Thus, our work aligns with the contemporary discussion on the application of machine learning in sepsis prognosis and significantly extends by highlighting our research’s novelty and clinical necessity. Firstly, our study was designed to investigate the correlation between immune-inflammation biomarkers and in-hospital mortality among septic patients based on machine learning algorithms and compared with the conventional logistic regression model and Sofa score. Secondly, by employing the XGBoost model coupled with SHapley Additive exPlanations (SHAP), we achieve superior predictive performance and enhance the interpretability of the model's outputs, making our findings directly actionable in clinical settings. This dual focus on accuracy and interpretability addresses a significant gap in the current literature, where many ML models remain black boxes. Lastly, to strengthen the model’s credibility, we recruited patients from three reputable medical centers, namely MIMIC-IV, eICU-CRD, and AmsterdamUMCdb, compared to single-center studies that have been prevalent in previous predictive models for sepsis [19, 23]. In addition, we meticulously excluded patients with HIV infection, rheumatic diseases, cancer or metastatic tumors, and hematological diseases to minimize potential bias associated with immunosuppression. Through advanced machine learning models, including the XGBoost model, we aimed to provide reliable prognostic insights into in-hospital mortality in septic patients.

## Methods

### Data source

Data for this study were obtained from the MIMIC-IV 1.0 database, the eICU-CRD 2.0, and the AmsterdamUMCdb 1.0.2. The MIMIC-IV 2.0 database, an updated version of MIMIC-III, comprises data from over 40,000 patients admitted to the ICU at the Beth Israel Deaconess Medical Center (BIDMC) [24]. The eICU-CRD contains data from multiple ICUs, having over 200,000 patients admitted in 2014 and 2015 [25]. The AmsterdamUMCdb contains approximately 1 billion clinical data points from 23,106 admissions of 20,109 patients [26].

Data collection and release were approved by the ethical standards of the institutional review board of the Massachusetts Institute of Technology (no. 0403000206) and complied with the Health Insurance Portability and Accountability Act (HIPAA).

### Participants

This study included participants aged 18 or older from the MIMIC-IV1.0, eICU databases 2.0, and AmsterdamUMCdb 1.0.2. Eligibility for inclusion was based on the following criteria: (1) Documented or suspected infection and a Sequential Organ Failure Assessment (SOFA) score of ≥ 2 according to the Sepsis-3.0 standards [3] in the first 24 h of ICU admission. (2) Documentation of peripheral complete blood count within the first 24 h of ICU admission.

Exclusion criteria included: (1) ICU stay of fewer than 24 h; (2) HIV infection, cancer, metastatic tumors, rheumatic diseases; (3) For patients with multiple hospitalizations, only the first ICU admission was considered for the study; (4) Total cholesterol, triglyceride, HDL, low-density lipoprotein (LDL) was not documented in the first 24 h.

### Data extraction, handling missing and outlier data

The following clinical information was extracted using Structured Query Language (SQL) statements: (1) Laboratory blood and biochemical examination within the first 24 h: WBC, platelets, neutrophil count, lymphocyte count, monocyte count, total cholesterol, HDL, LDL, blood glucose. (2) Demographics and vital signs within the first 24 h: age, sex, heart rate, systolic blood pressure, diastolic blood pressure, temperature (℃), and respiratory rate. (3) Blood gas analysis within the first 24 h: arterial partial pressure of oxygen (PaO_2_), arterial partial pressure of carbon dioxide (PaCO_2_). (4) ICU details: the length of ICU stays and the inpatient survival status. (5) Comorbidity and treatment modalities: myocardial infarction, congestive heart failure, chronic pulmonary, liver disease, renal disease, mechanical ventilation, and dialysis. In cases where a variable was recorded multiple times within the first 24 h of ICU admission, the value associated with the greatest severity of illness was used. The NLR was computed as the ratio of neutrophils to lymphocytes, and the LMR was calculated as the ratio of lymphocytes to monocytes. The PLR was calculated from the ratio of platelets to lymphocytes. The MHR was calculated from the ratio of monocytes to HDL. The NHR was calculated from the ratio of neutrophils to HDL.

Variables missing for over 30%, including PaO_2_fio_2_ratio, Fio_2_, Lactate,Spo_2_, Paco_2_, Pao_2_, Ph, LDL, were excluded from analysis (Additional file 1: Fig. S1). The remaining 45 predictor candidates measured at the ICU admission were selected for further analysis. Multiple imputations utilizing predictive mean matching (pmm) with the "mice" package imputed missing values for selected variables [27]. Random forest outlier detection was implemented (Additional file 2: Fig. S2), with outliers replaced by pmm using the outForest R package [28, 29].

### Statistical analysis

We utilized SQL statements to extract the required clinical information. All analyses were carried out using R4.0.5. Continuous variables were represented as the mean ± SD or median (interquartile) and compared using Student's t test for normally distributed variables or the Mann–Whitney U test for non-normally distributed variables. Categorical variables were expressed as proportions and analyzed using the Chi-square or Fisher's exact tests. The participants in the survey were randomly divided into three different cohorts: the training cohort, the validation cohort, and the test cohort. The training cohort comprised 48% of the total participants (*n* = 1696) and was used to identify essential features. Meanwhile, the validation cohort (*n* = 1132), which accounted for 32% of the participants, was used to fine-tune the hyperparameters and identify the most effective classifiers. Finally, the test cohort, which made up 20% of the total number of participants (*n* = 707), was used to evaluate the performance of the selected features and classifiers. LASSO regularization was employed for variable selection, identifying pertinent variables while disregarding others to reduce model complexity and mitigate overfitting risks [30, 31]. A vital advantage of this approach is facilitating model interpretability by enhancing the understanding of underlying relationships. Ten-fold cross-validation with the "glmnet" package estimated optimal penalty parameters (lambda) and beta coefficients for selected variables in the training cohort [32]. This rigorous cross-validation process ensured robustness in model selection and parameter estimation. We used the glmnet package for LASSO regression, setting the alpha parameter to 1. As part of the procedure, it automatically normalizes the data. In addition, we performed outlier preprocessing to improve data quality and employed tidymodel package to normalize the data with Z-scores after handling outliers for validation and test cohorts. Therefore, no further normalisation of the dataset is required for machine learning modelling. A comprehensive ensemble of seven machine learning models, including eXtreme Gradient Boosting (XGBoost), logistic regression (LR), random forest (RF), support vector machine (SVM), K Nearest Neighbor (KNN), Naive Bayes, and Decision Tree (DT), estimated the predictive models in our study. Model discriminative accuracy was evaluated using the area under the receiver operating characteristic curve (AUC-ROC), a widely accepted metric, F1 scores, precision, and recall. Decision curve analysis (DCA) quantified net benefit across varying threshold probabilities to further assess the practical utility and potential clinical impact, providing crucial insights into model clinical relevance and optimal decision strategies based on predictive outcomes [33]. Ten-fold Cross-Validation was applied to the validation set to reduce the bias. Spearman correlation, Pearson correlation analysis, distance correlation, mutual information, and maximal information coefficient examined the associations among the continuous predictor variables. Restricted cubic splines (RCS) with strategic knot positioning (the 5th, 35th, 65th, and 95th percentiles) explored potential non-linear relationships between continuous risk factors using the Regression Modeling Strategies (rms) package in R [34]. Multivariate adjustment in RCS analyses helps control for these variables' effects and get a more accurate estimate of the relationship between the independent variable and the in-hospital mortality. Collectively, these rigorous statistical techniques ensured robust and reliable results. The machine learning models were performed based on the “tidymodels” R package. The R package “shapviz” was used to evaluate the SHAP value and visulize the feature importance of XGBoost model.

## Results

### Clinical characteristics and demographics of patients

**Fig. 1:**
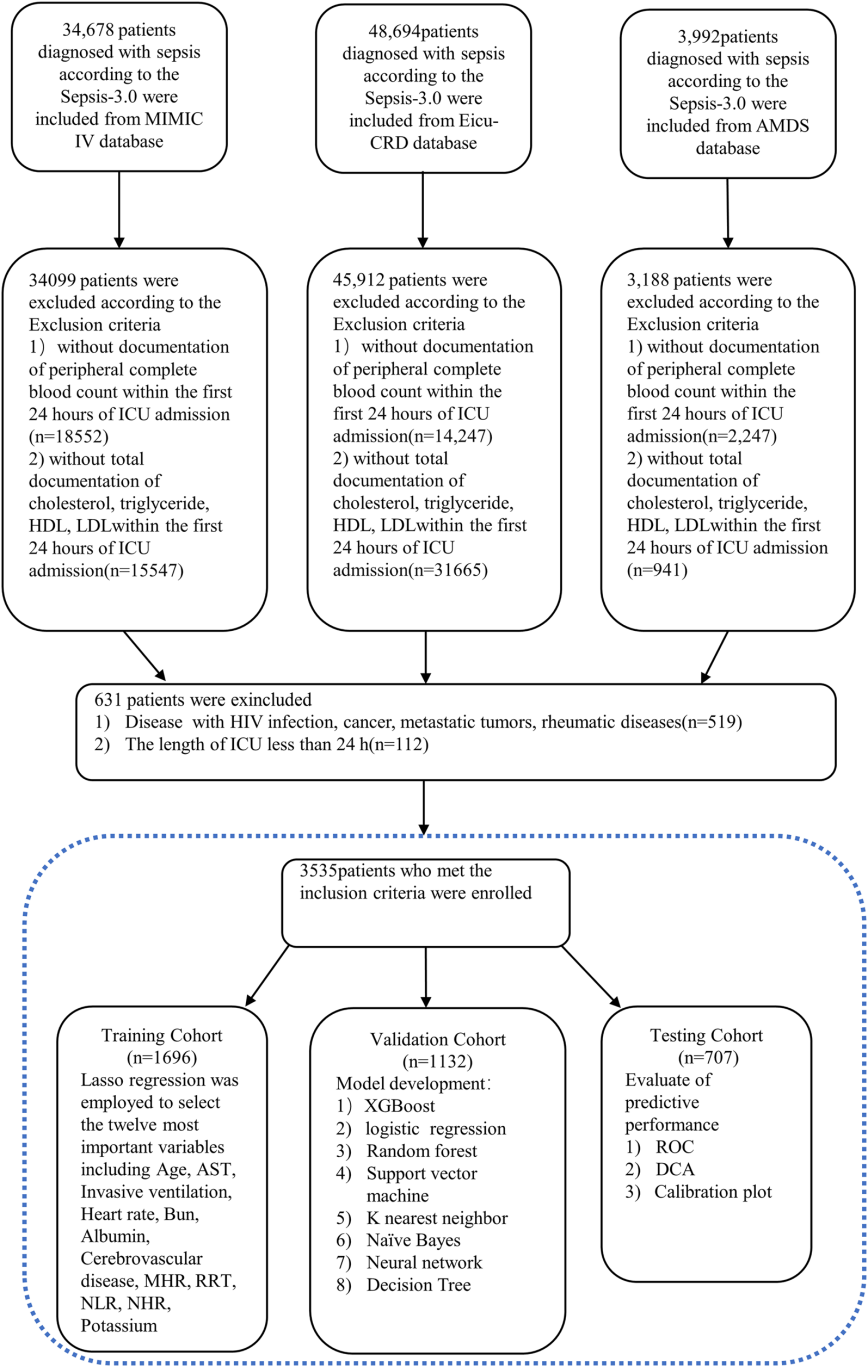
A flowchart illustrating the regulatory model of patient enrollment and analysis workflow. Following the exclusion of 83,829 patients, 3535 patients were included from three databases. MIMIC-IV database: Medical Information Mart for Intensive Care-IV database, eICU-CRD: eICU Collaborative Research Database; AMDS: Amsterdam University Medical Centers database; ROC: receiver operating characteristic curve; DCA: Decision curve analysis

3535 patients meeting the inclusion criterion were ultimately recruited for this study (Fig. 1). The median participant age was 66 years (IQR, 55–77 years), with 1977 of 3535 (56%) being male (Table 1). Diabetes was the most common comorbidity (1116 of 3535, 31.6%), followed by congestive heart failure (680 of 3535, 19.2%). Non-survivors tend to be older (64.0 [53.0–75.0] vs. 69.0 [58.4–80.0], *p* < 0.01) and exhibited greater vulnerability to medical interventions, including invasive ventilation (79.1% vs. 59.8%, *p* < 0.01) and renal replacement treatment (RRT) (19.3% vs. 8.3%, *p* < 0.01). The median value of HDL, lymphocytes, hemoglobin, albumin, and LMR was higher in survivors, while the inflammatory biomarkers, including NLR and NHR, were significantly lower than in the non-survivors.

**Table 1:** Baseline characteristics of the patients

	Survivors	Non-survivors	*P* value
	*N* = 2980	*N* = 555	
Age, years	65.(0 55.0–76.0)	72.0 (62.1–80.0)	< 0.001
Gender			0.260
F	1326 (44.5%)	232 (41.8%)	
M	1654 (55.5%)	323 (58.2%)	
White blood cells, × 10^3^/µL	13.6 (10.1–18.4)	14.7 (10.6–20.0)	0.001
Lymphocytes	1.45 (0.90–2.31)	1.26 (0.79–2.12)	0.001
Neutrophils	10.9 (7.44–15.2)	11.9 (7.85–16.9)	0.002
Monocytes	0.93 (0.59–1.42)	0.94 (0.55–1.42)	0.588
Platelet	225 (169–296)	214 (154–282)	0.004
Triglycerides, mg/dL	106 (74.0–157)	104 (77.0–147)	0.757
Total cholesterol, mg/dL	128 (99.0–164)	114 (88.9–150)	< 0.001
High-density lipoprotein, mg/dL	35.0 (25.0–47.0)	32.0 (21.5–44.0)	< 0.001
Hemoglobin, g/dL	12.6 (10.9–14.3)	12.4 (10.3–14.0)	0.011
Albumin, g/dL	3.20 (2.70–3.70)	3.00 (2.40–3.50)	< 0.001
Bun, mg/dL	25.0 (16.2–40.0)	34.0 (20.9–51.0)	< 0.001
Calcium, mg/dL	8.70 (8.20–9.20)	8.70 (8.10–9.20)	0.025
Creatinine, mg/dL	1.25 (0.90–2.00)	1.70 (1.10–2.60)	< 0.001
Glucose, mg/dL	171 (133–229)	187 (143–247)	< 0.001
Sodium, mmol/L	140 (138–143)	141 (138–146)	< 0.001
Potassium, mmol/L	4.40 (4.10–4.90)	4.60 (4.20–5.20)	< 0.001
ALT, IU/L	30.0 (19.0–55.2)	38.0 (21.0–97.0)	< 0.001
AST, IU/L	36.0 (23.0–76.0)	60.0 (32.0–200)	< 0.001
PH	7.41 (7.36–7.46)	7.40 (7.35–7.46)	0.091
Heart rate	106 (92.0–122)	113 (96.0–129)	< 0.001
Systolic blood pressure	155 (136–176)	155 (134–178)	0.898
Diastolic blood pressure	90.0 (77.0–105)	91.0 (76.0–105)	0.631
Mean blood pressure	162 (137–187)	160 (134–186)	0.306
Respiratory rate	28.0 (24.0–35.0)	30.0 (25.0–37.0)	0.001
Temperature	37.4 (37.0–38.0)	37.3 (36.9–37.9)	0.072
First day sofa score	6.00 (4.00–9.00)	9.00 (7.00–12.0)	< 0.001
Length of hospital stays	38.0 (14.0–86.0)	36.0 (8.00–118)	0.605
Renal replacement therapy			< 0.001
No	2732 (91.7%)	448 (80.7%)	
Yes	248 (8.32%)	107 (19.3%)	
Invasive ventilation			< 0.001
No	1197 (40.2%)	116 (20.9%)	
Yes	1783 (59.8%)	439 (79.1%)	
Myocardial infarct			0.293
No	2648 (88.9%)	484 (87.2%)	
Yes	332 (11.1%)	71 (12.8%)	
Congestive heart failure			0.838
No	2409 (80.8%)	446 (80.4%)	
Yes	571 (19.2%)	109 (19.6%)	
Peripheral vascular disease			0.566
No	2834 (95.1%)	524 (94.4%)	
Yes	146 (4.90%)	31 (5.59%)	
Dementia			0.835
No	2831 (95.0%)	529 (95.3%)	
Yes	149 (5.00%)	26 (4.68%)	
Chronic pulmonary disease			0.465
No	2533 (85.0%)	479 (86.3%)	
Yes	447 (15.0%)	76 (13.7%)	
Peptic ulcer disease			0.612
No	2933 (98.4%)	544 (98.0%)	
Yes	47 (1.58%)	11 (1.98%)	
Renal disease:			0.003
No	2508 (84.2%)	438 (78.9%)	
Yes	472 (15.8%)	117 (21.1%)	
diabetes:			0.003
No	2009 (67.4%)	410 (73.9%)	
Yes	971 (32.6%)	145 (26.1%)	
Liver disease:			0.015
No	2876 (96.5%)	523 (94.2%)	
Yes	104 (3.49%)	32 (5.77%)	
Cerebrovascular disease:			0.010
No	2509 (84.2%)	442 (79.6%)	
Yes	471 (15.8%)	113 (20.4%)	
LMR	1.63 (1.00–2.56)	1.42 (0.94–2.48)	0.018
NLR	7.16 (4.18–12.0)	8.71 (4.90–14.7)	< 0.001
PLR	152 (94.5–238)	151 (90.5–269)	0.436
MHR	0.03 (0.02–0.05)	0.03 (0.01–0.05)	0.204
NHR	0.31 (0.19–0.50)	0.35 (0.21–0.64)	< 0.001

LMR**:** the ratio of lymphocytes to monocytes; NLR**:** the ratio of neutrophils to lymphocytes; PLR**:** the ratio of platelets to lymphocytes; MHR**:** the ratio of monocytes to high-density lipoprotein; NHR**:** the ratio of neutrophils to high-density lipoprotein

### Model development and validation

**Fig. 2:**
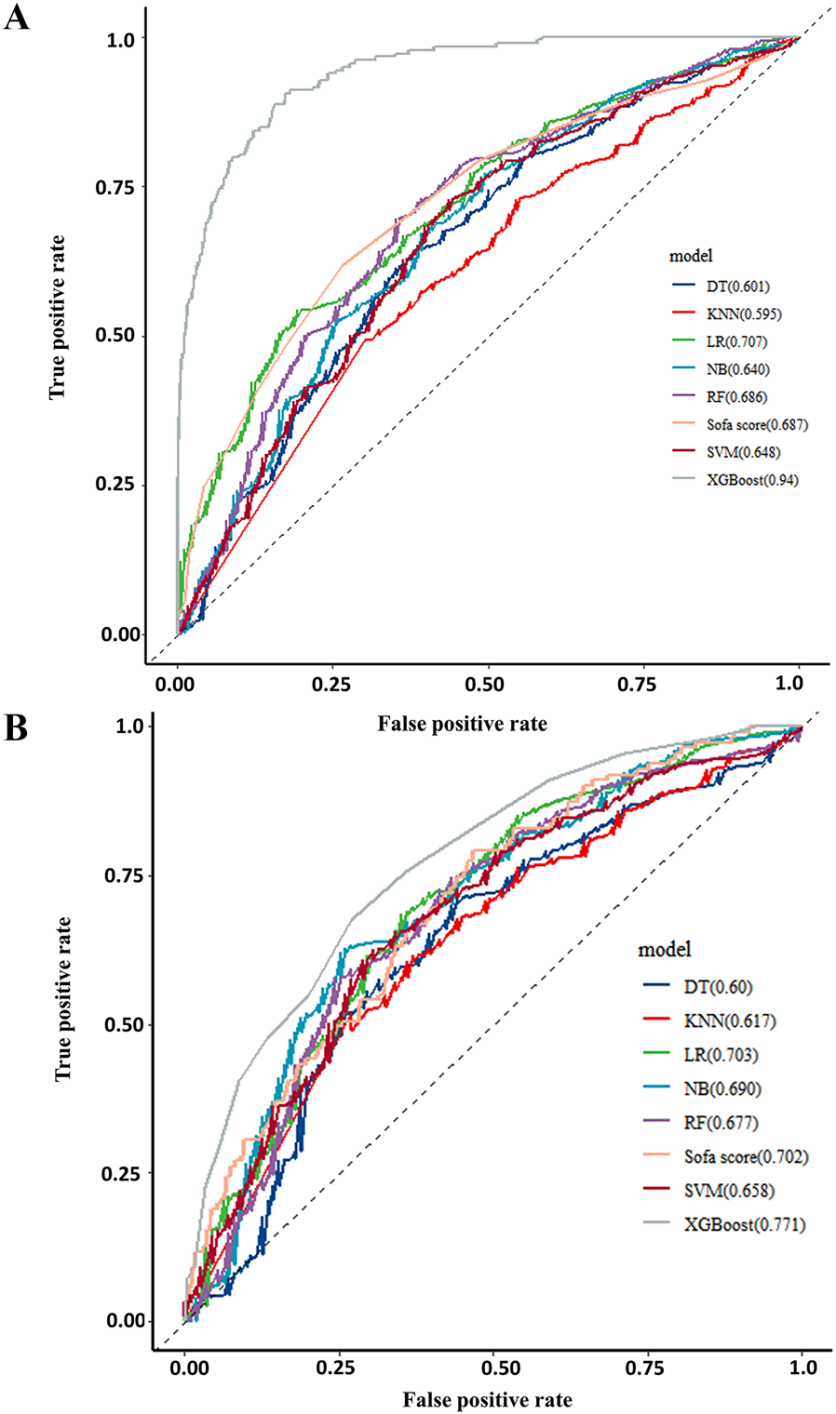
The ROC curve comparison of six models and Sofa score in training cohort and validation cohort. DT: Decision Tree; XGBoost: eXtreme Gradient Boosting; KNN: k-Nearest Neighbors; RF: Random Forest; NB: Naive Bayes; LR: Logistic Regression; SVM: Support Vector Machine. **A **The ROC curve of validation Cohort, **B **The ROC curve of test Cohort

**Fig. 3:**
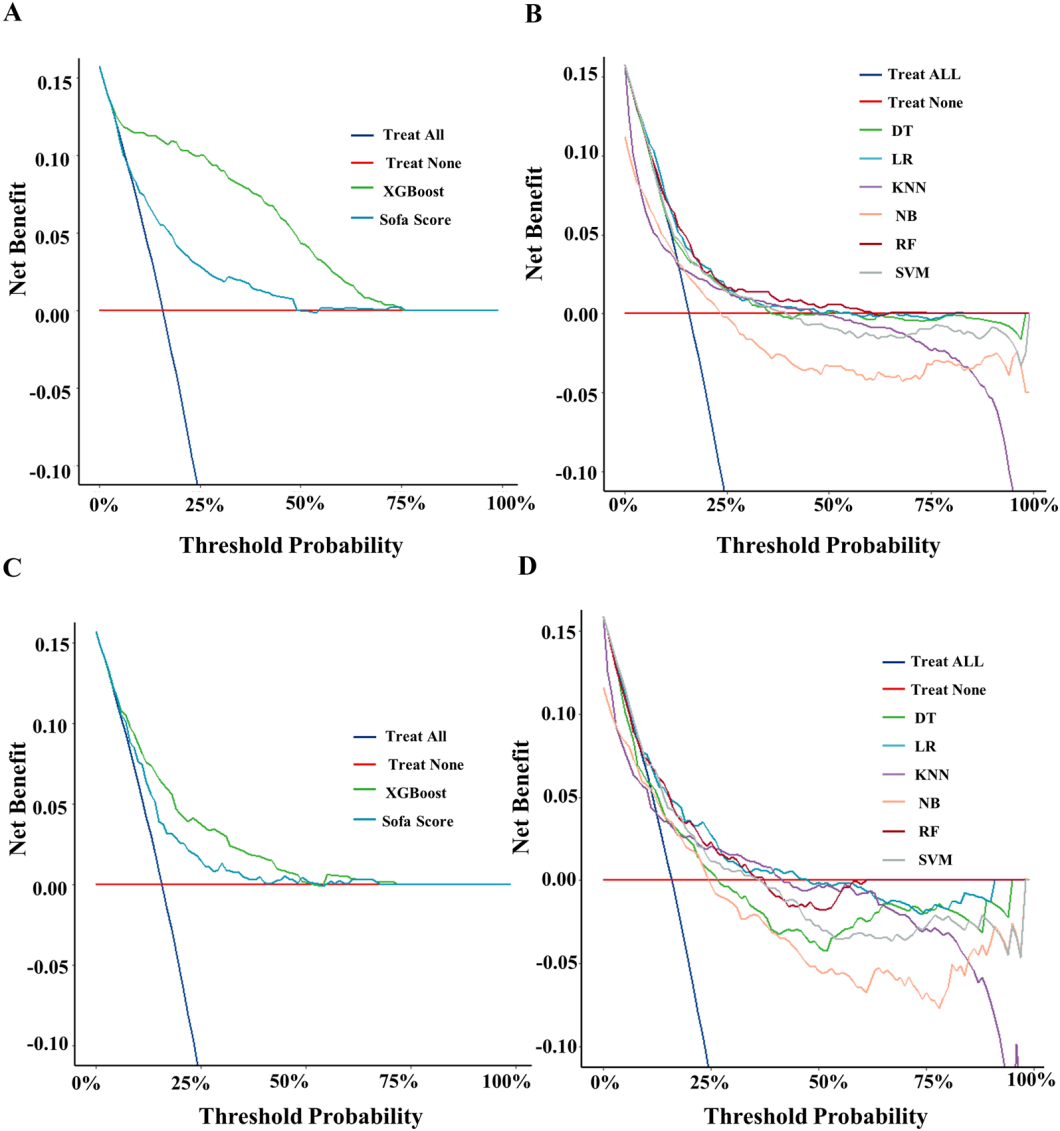
The DCA curve comparison of six models and Sofa score in training cohort and validation cohort. DCA: Decision curve analysis; DT: Decision Tree; XGBoost: eXtreme Gradient Boosting; KNN:k-Nearest Neighbors; RF: Random Forest; NB: Naive Bayes; LR: Logistic Regression; SVM: Support Vector Machine. **A **DCA curve of XGBoost and Sofa score in validation Cohort. **B** DCA curve of other six models in validation Cohort. **C** DCA curve of XGBoost and Sofa score in Validation Cohort.** D** DCA curve of other six models in test Cohort

A total of 45 clinical variables were collected according to the inclusion criteria. LASSO regression for the training cohort identified 12 variables associated with sepsis prognosis out of 45 clinical parameters (Additional file 3: Fig. S3): Age, Heart rate, AST, invasive ventilation treatment, renal replacement treatment, albumin, cerebrovascular disease, MHR, NLR, NHR, BUN, and Potassium. Seven ML binary classifiers were constructed to predict sepsis mortality risk based on the selected variables: XGBoost, Random Forest (RF), Naive Bayes (NB), Logistic Regression (LR), Support Vector Machine (SVM), k-Nearest Neighbors (KNN), and Decision Tree (DT). In the validation cohort, XGBoost demonstrated superior model fit with an area under the curve (AUC) of 0.94 and an F1 score of 0.937 compared to a Sofa score AUC of 0.687 and an F1 score of 0.914 (Fig. 2A). The optimal hyperparameters for the XGBoost model: learning_rate = 0.003, tree_depth = 8, subsample = 0.876, min_child_weight = 8, and n_estimators = 1024. Compared with XGBoost model, the other models also showed comparatively lower efficiency in AUC and other indices (AUC: RF, 0.686, NB, 0.640; LR, 0.707 SVM, 0.648; KNN, 0.595; DT,0.601; F1 score: RF, 0.917, NB,0.914; LR, 0.915; SVM,0.881; KNN,0.892; DT, 0.871) (Table 2 and Additional file 6: Table S1). This trend persisted in the test cohort (Fig. 2B). Given its optimal performance, the XGBoost model was selected for further prediction. DCA also shows that the XGBoost model conferred more significant clinical benefit across threshold probability (0.25) versus the Sofa score and other models in the validation and test cohorts (Fig. 3). Additionally, calibration curve analysis revealed superior XGBoost model goodness-of-fit over SOFA scoring in the test cohort (Additional file 4: Fig. S4).

**Table 2:** Performances of the seven machine learning models and Sofa score for predicting in-hospital mortality

Model	AUC	Precision	Recall	F1 Score
XGBoost	0.94	0.882	0.918	0.937
Sofa score	0.687	0.849	0.879	0.914
Logistic regression	0.707	0.850	0.878	0.915
Random forest	0.686	0.852	0.882	0.917
K-nearest Neighbor	0.622	0.855	0.873	0.892
Naïve Bayes	0.590	0.842	0.876	0.914
SVM	0.648	0.855	0.873	0.892
Decision Tree	0.595	0.853	0.861	0.871

XGBoost: extreme Gradient Boosting, SVM: Support Vector Machine, AUC: the area under curve

### Model explanation

**Fig. 4:**
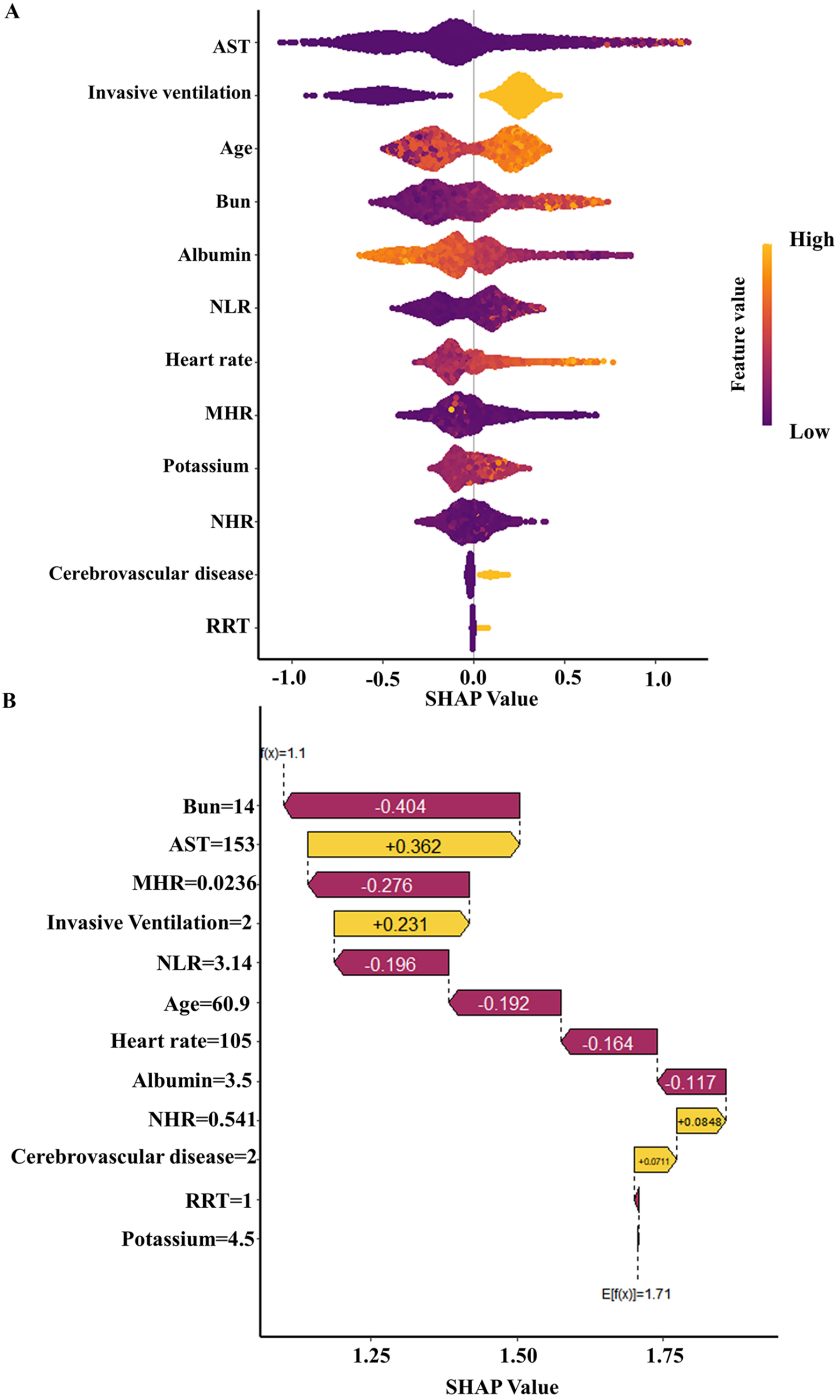
**A** Scatter plot of feature values and SHAP values. The purple part of the feature value represents a lower value. **B** Consent waterfall plot showing an example of interpretability analysis for a patient. The yellow part of the feature value represents a positive effect on the model. The deep red part of the feature value represents a represents a negative effect on the model

**Fig. 5:**
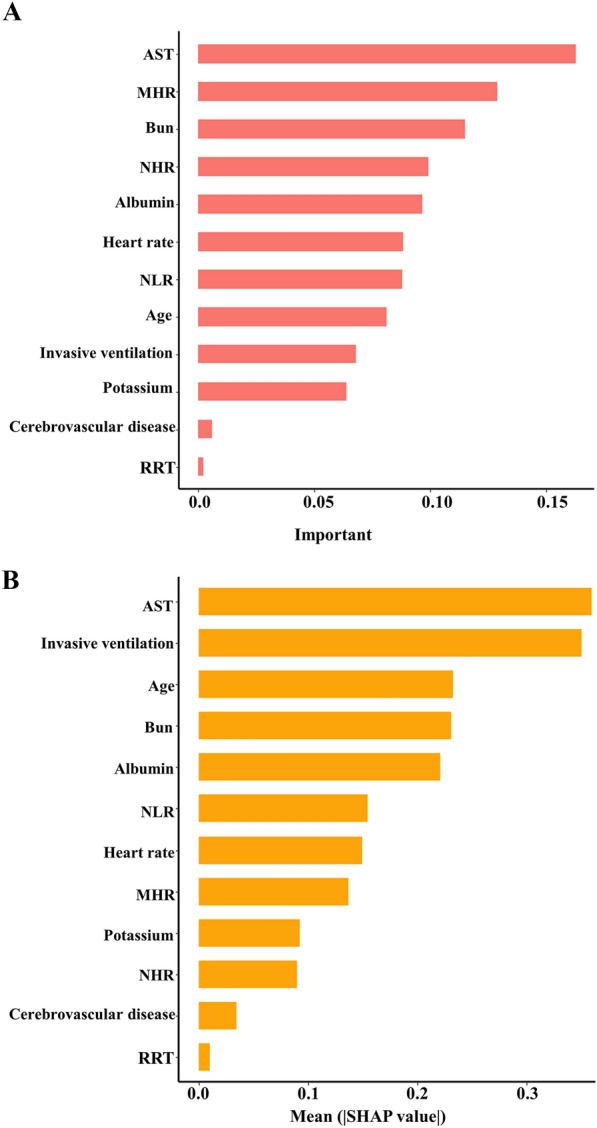
The feature importance of SHAP method and conventional method for XGBoost model. **A **Feature importance of conventional method for the XGBoost model. **B **Feature importance of SHAP method for the XGBoost model. BUN: Urea nitrogen

**Fig. 6:**
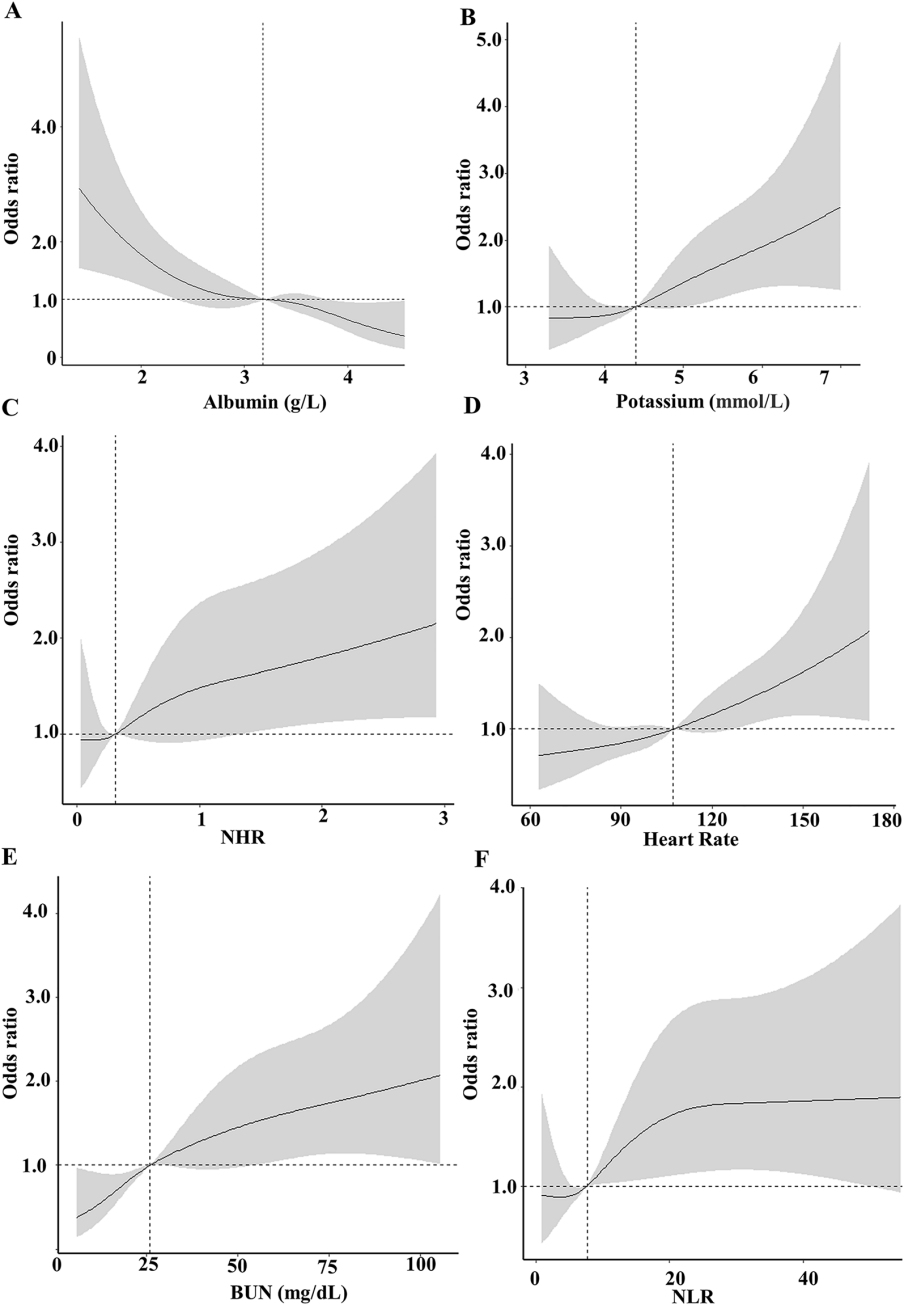
The association between variables and hospital mortality. Albumin (**A**), Potassium (**B**), NHR (**C**), Heart rate (**D**), BUN (**E**), NLR (**F**): the restricted cubic splines with four knots. The horizontal dashed line represents the reference OR of 1.0. The model was multivariate-adjusted for Age, AST, whether or not invasive ventilation treatment, whether or not renal replacement treatment, Albumin, whether or not have cerebrovascular disease, MHR, NLR, NHR, Potassium. OR odds ratio; 95% CI 95% confidence interval

**Fig. 7:**
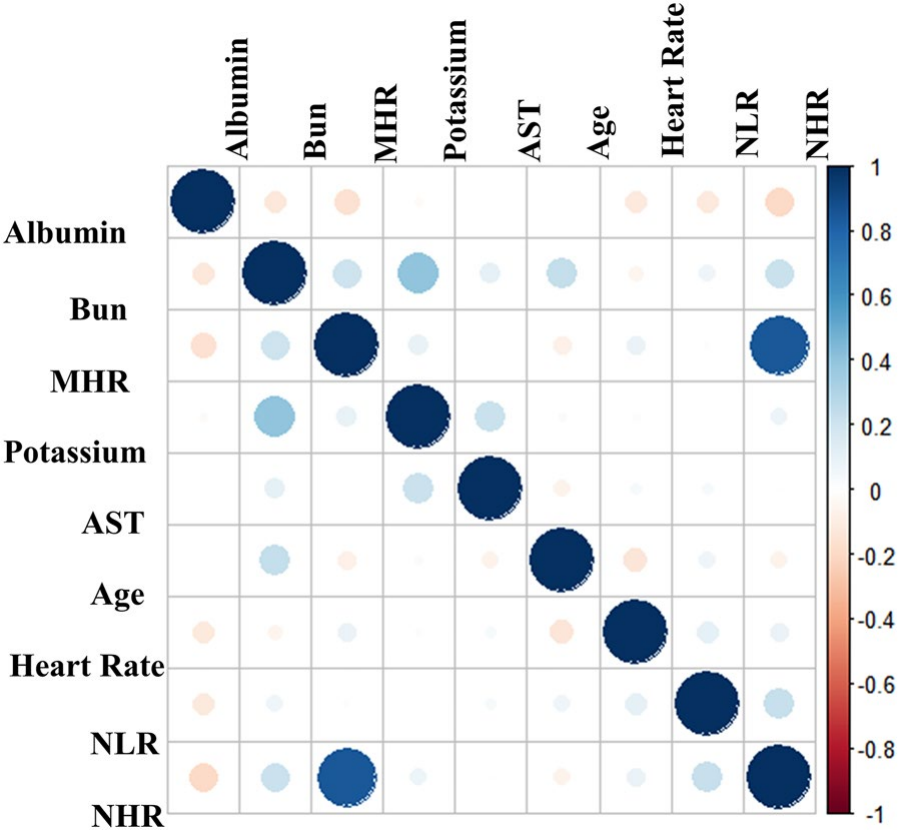
Spearman correlation analysis between variables. The color spectrum, ranging from blue to yellow, represents the degree of correlation: closer to blue indicates a stronger positive correlation, while closer to yellow indicates a stronger negative correlation

SHAP (SHapley Additive exPlanations) is a versatile method capable of elucidating the foundations of machine learning models. It uses the principle of optimal credit allocation, rooted in the Shapley value, to measure the importance of features through a game-theoretic lens, thereby subtly navigating the opacity often associated with machine learning models and achieving consistent interpretability [35]. One of the unique strengths of SHAP, particularly in global interpretation, is its ability to reveal the importance of features and describe their outputs in relationship to their interactions. Our study applied SHAP values specifically for interpreting the XGBoost model. As illustrated in Fig. 4A, the horizontal positioning in the SHAP plot illustrates whether eigenvalues are associated with a higher or lower predictive tendency. At the same time, the color spectrum indicates whether the variable is high (indicated in purple) or low (indicated in yellow) for a particular observation. Notably, elevated age, blood potassium levels, neutrophil-to-lymphocyte ratio (NLR), heart rate, and invasive ventilation treatment were observed to influence and thus drive mortality prediction positively. Conversely, increases in serum albumin negatively impacted the prediction of survival. These findings are consistent with established medical principles and confirmed by previous studies [7, 36, 37], thus enhancing our model's credibility and explanatory veracity. SHAP values can provide valuable insights to help physicians interpret individual predictions [38]. Additionally, we generate a waterfall plot visualizing the SHAP values for a given patient (Fig. 4B), with features ranked from most to least important. This would allow the physician to quickly understand which factors are most strongly associated with an increased or decreased risk of septic death for that patient. Furthermore, a comparative analysis of the results obtained using the traditional XGBoost feature importance method and SHAP was conducted. Firstly, feature importance was calculated using the in-built functionality of the XGBoost algorithm. As shown in Fig. 5A, the results are presented visually in bar charts, which rank the features according to their contribution to the model. The analysis revealed six main features, AST, MHR, BUN, NHR, albumin, and age, which differed from the results of SHAP (Fig. 5B) and weakened the prognostic impact of mechanical ventilation on in-hospital mortality [39, 40]. The difference arises from their distinct analytical approaches. SHAP values provide a local, accurate interpretation of how each feature influences individual predictions, considering feature interactions. XGBoost feature importance, in contrast, gives a global view of feature contributions across the entire model [41], and SHAP values are particularly adept at capturing non-linear effects and interactions between features, which might not be fully represented by traditional feature importance metrics in XGBoost42. SHAP appears more interpretable than traditional XGBoost feature significance methods, providing a more comprehensive assessment of feature significance. The sophisticated understanding provided by SHAP helps to outline the intricate relationships between features and model outputs, thereby increasing the interpretive transparency of machine learning models. Multivariate-adjusted restricted cubic splines further explored variables' relationships with in-hospital mortality. Liner associations were found for NLR, NHR, Potassium, heart rate, BUN, and albumin (*p* for non-linear > 0.05) (Fig. 6A–F). However, non-linear relationships between in-hospital mortality and MHR, Age, and AST were observed. A U-shaped association exists for MHR, with higher and lower values conferring greater in-hospital mortality risk than the curve bottom (0.028) (Additional file 5: Fig. S5A). Age and AST demonstrated steep initial increases, plateauing at certain levels (AST: 234IU/L, Age: 78 years) (Additional file 5: Fig. S5B, C). In addition, A statistically significant positive correlation was observed between NHR and MHR, BUN (*p* < 0.05), as depicted in Fig. 7, and the results from Pearson correlation, distance correlation, mutual information, and maximal information coefficient analyses align with the trends observed in our Spearman correlation (Additional file 7: Table S2, Additional file 8: Table S3, Additional file 9: Table S4, Additional file 10: Table S5). However, the relationships of NHR and BUN to in-hospital mortality (Fig. 6C and E) differ from SHAP analysis. The key point here is that SHAP values reflect not solely the correlation between features but also how each feature contributes to the model's prediction for each specific instance, meaning that the SHAP value for a feature in a given prediction is an average of its marginal contribution across all possible combinations of features, which can lead to differences in SHAP values for correlated features [43]. In summary, while NHR and BUN are correlated, their different SHAP values in our model reflect the complex interactions and the unique contribution of each feature within the context of the model's predictions. This discrepancy in SHAP values underscores the complexity of the feature interactions within the model and does not necessarily contradict the observed correlation between the features.

## Discussion

The phenomenon of missing data is a common problem in most research endeavors and can significantly impact the validity of data inferences and reduce the sample size when restricted to analyses of complete cases [44, 45]. We chose multiple imputation as our strategy for dealing with missing data, because it is more likely to provide more accurate estimates than other methods, such as mean imputation or listwise deletion, even at the cost of increased analytical complexity [46]. The procedures for dealing with missing data were carefully designed in collaboration with clinicians familiar with the study population and the data collection process. Their input was sought to determine the most appropriate approach to missing data management, including identifying variables to be included in the estimation model. In this retrospective study utilizing three large-scale public ICU databases, we developed and validated seven machine-learning algorithms to predict the in-hospital mortality of patients with sepsis. The XGBoost model outperformed LR, RF, NB, KNN, DT, and SVM. Furthermore, the XGBoost model demonstrated superior performance compared to traditional Sofa scores. XGBoost is well-suited for capturing complex non-linear relationships between features without the extensive data preprocessing required by deep learning models. Considering the computational resources available, XGBoost offers a more scalable and less resource-intensive alternative to deep learning models, which often require significant computational power and data volume to achieve optimal performance [47–52]. In critical care research, XGBoost has been extensively utilized to predict the in-hospital mortality of patients and may assist clinicians' decision-making [20, 53]. SHAP values offer insight into how each feature influences the model's prediction, providing interpretability support in understanding the model's decision-making process, fostering trust, and facilitating the model's adoption in clinical practice [54, 55]. We employed SHAP to explain the XGBoost model to ensure model performance and clinical interpretability, which enables physicians to comprehend the model's decision-making process better and facilitates the utilization of prediction results.

The most impactful parameters contributing to predicted mortality risk in sepsis patients were Age, AST, invasive ventilation treatment, and BUN. NLR and serum albumin were also highly predictive of in-hospital mortality in ICU sepsis patients, consistent with previous research [56, 57]. Interestingly, some inflammatory biomarkers, such as NHR and MHR, critically impacted hospital mortality of sepsis patients in the XGBoost model. Previous prognostic prediction models utilizing inflammatory biomarkers have been developed, such as a nomogram by Chen et al. based on age, NLR, PLR, LMR, and RDW to predict 28-day mortality in sepsis [58]. Li et al. [17] were the first to develop an XGBoost model incorporating inflammatory biomarkers like NLR, NHR, and MHR, demonstrating the combination of NLR and MHR as an independent risk factor with predictive capability for 28-day mortality in patients with sepsis. Our model encompassed three ICU databases to improve credibility and generalizability compared to previous single-center models. An observational study in Australia and New Zealand also demonstrated sepsis mortality under 5% without comorbidities or advanced age [59]. Consistent with the findings, our analysis also demonstrated that comorbidities like cerebrovascular disease contributed to higher sepsis mortality. Our initial exclusion of patients with HIV, rheumatic disease, cancer, or metastatic tumors minimized potential immunosuppression-related biases across the three databases. To evaluate the performance of our proposed approach, we use the Sequential Organ Failure Assessment (SOFA) score as a baseline comparison. The SOFA score is a validated tool for assessing morbidity in critical illness and is commonly used for benchmarking in observational studies [60]. While the SOFA score has been used for over 25 years, it remains a relevant metric for objectively describing patterns of organ dysfunction in critically ill patients. For example, an increase in the SOFA score, such as requiring renal replacement therapy, has been associated with higher overall ICU mortality [61]. Our study aims to complement the SOFA score by incorporating inflammatory biomarkers and machine learning techniques to improve risk prediction in sepsis patients. We believe supplementing the SOFA score in this manner may provide unique and clinically meaningful information. However, several limitations exist. First, our current study has limitations in fully establishing external validity and may be subject to bias, even with internal ten-fold cross-validation. Secondly, our data were sourced from the initial 24 h of ICU admission. The omission of dynamic changes in inflammatory markers in our study might limit our ability to capture the full spectrum of immunological changes in sepsis. The work of Reyna et al. demonstrates how machine learning can uncover hidden patterns in vital signs, enhancing sepsis outcome predictions [62]. Similarly, Nesaragi highlights the importance of incorporating ratios and higher order interactions among vital signs, a methodological approach that aligns with our study's efforts to improve predictive accuracy [49]. These references underscore the potential of leveraging complex statistical relationships to predict better sepsis outcomes, a direction our research aims to develop further. The limitation posed by the lack of comprehensive time-series data restricts our ability to capture the dynamism inherent in sepsis progression and limits the utility score's application, reflecting a broader challenge in the field. The utility score's emphasis on the clinical impact of predictions necessitates detailed data, underscoring the need for future studies to access more granular clinical information [50–52, 63–66]. Future research should incorporate longitudinal analyses to model better the temporal variations in inflammatory markers, a direction underscored by the referenced studies' success in utilizing such data for enhanced prediction models. Thirdly, the retrospective nature of our study introduces inherent selection bias and machine learning, where the nature of the input data constrains the model's output, focusing more on correlation and less on the underlying causal mechanisms [67]. Therefore, a well-designed prospective study is essential to validate the model's utility. Fourthly, limited by the MIMIC-IV, eICU-CRD databases, and AmsterdamUMCdb, critical data such as temporal dynamics of inflammatory biomarkers were insufficiently recorded, hindering analysis. Despite these limitations, we hope our constructed model will assist clinicians in the timely treatment of ICU sepsis patients. In our subsequent research, we aim to include dynamic markers to capture the evolving nature of sepsis more effectively, thereby contributing to a more robust and nuanced understanding of the disease. We will focus on continuous modeling of predictors in sepsis research, reducing reliance on dichotomization to minimize associated potential errors. Additionally, we acknowledge our current study's limitations regarding the establishment of external validity and recognize the need for further validation through prospective multi-center studies. We plan to explore the model's applicability to different patient populations and clinical settings, aiming to improve the model's predictive accuracy and external validity continuously. In line with these efforts, we are establishing our sepsis database to further validate our model's external validity.

## Conclusion

In conclusion, this study demonstrates that machine learning models integrating inflammatory biomarkers can significantly improve the prediction of the risk of in-hospital mortality among sepsis patients. The XGBoost model outperformed traditional scoring systems and other machine learning algorithms, with an AUC of 0.94 and an F1 score of 0.937 in predicting in-hospital mortality. Specifically, the most significant determinants included increased levels of AST and BUN, advanced age, elevated NLR, and the requirement for invasive ventilation. The model provides a robust method to rapidly stratify patients upon ICU admission and could guide clinical decisions. We also hope the model could serve as a supplementary tool to the SOFA score in this manner and may provide unique and clinically meaningful prognostic information beyond what is captured by the SOFA score alone.

### Supplementary Information

**Additional file 1: Figure S1.** Percentages of missing data for all included variables.

**Additional file 2: Figure S2.** Count of outliers for all eligible variables

**Additional file 3: Figure S3.** Feature selection using the LASSO regression model. (A) Tuning parameter (λ) selection in the LASSO model used tenfold cross-validation via minimum criteria. LASSO, least absolute shrinkage and selection operator. (B) LASSO coefficient profiles of the 45 baseline features.

**Additional file 4: Figure S4.** Calibration curve for assessing the goodness of fit for XGBoost model and SOFA score in validation Cohort. **A:** Calibration curve of XGBoost model in validation cohort. **B:** Calibration curve of Sofa scores in validation cohort.

**Additional file 5: Figure S5.** Multi variable-adjusted odds ratios for association between hospital mortality and MHR (A), AST (B), and Age (C).

**Additional file 6: Table S1.** Performances of the seven machine learning models and Sofa score for predicting in-hospital mortality from test cohort.

**Additional file 7: Table S2.** The Pearson correlation between variables.

**Additional file 8: Table S3.** The distance correlation between variables.

**Additional file 9: Table S4.** The mutual information between variables.

**Additional file 10: Table S5.** The maximal information coefficient between variables.

## Data Availability

The MIMIC-2.0 dataset is accessible on PhysioNet (https://physionet.org/content/mimiciv/2.0/). The eICU-CRD dataset is also available on PhysioNet(https://physionet.org/content/eicu-crd/2.0/).Additionally,the AmsterdamUMCdb is available through Amsterdam Medical Data Science (https://amsterdammedicaldatascience.nl/amsterdamumcdb/).
