# Supplementary figures and images for: Predicting sepsis in-hospital mortality with machine learning: a multi-center study using clinical and inflammatory biomarkers
Source: Eur J Med Res. 2024 Mar 6;29:156. doi: 10.1186/s40001-024-01756-0 (PMC10918942; doi:10.1186/s40001-024-01756-0)

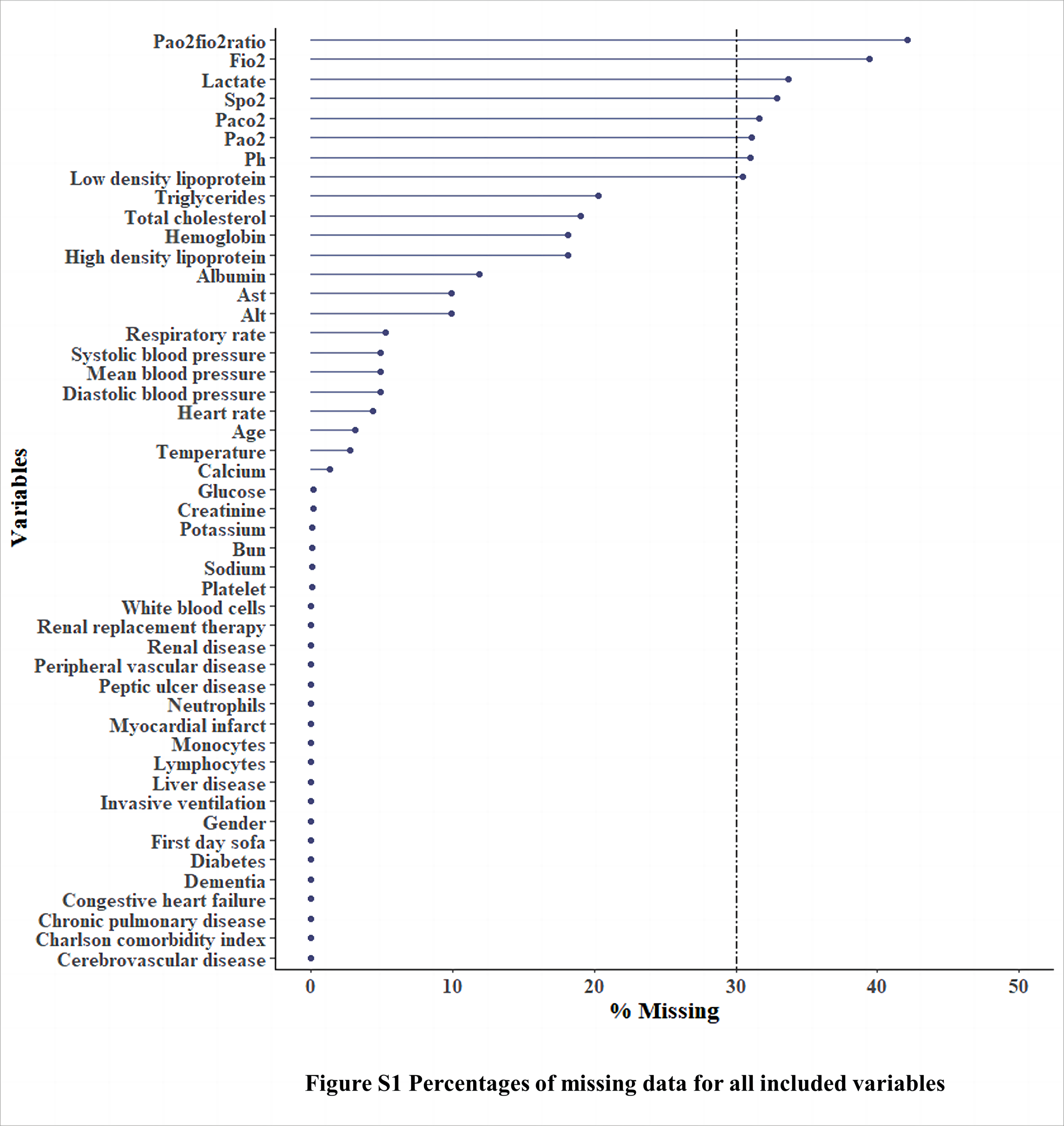

Supplement: Supplementary file 1 — Additional file 1: Figure S1. Percentages of missing data for all included variables. [file 40001_2024_1756_MOESM1_ESM.png]

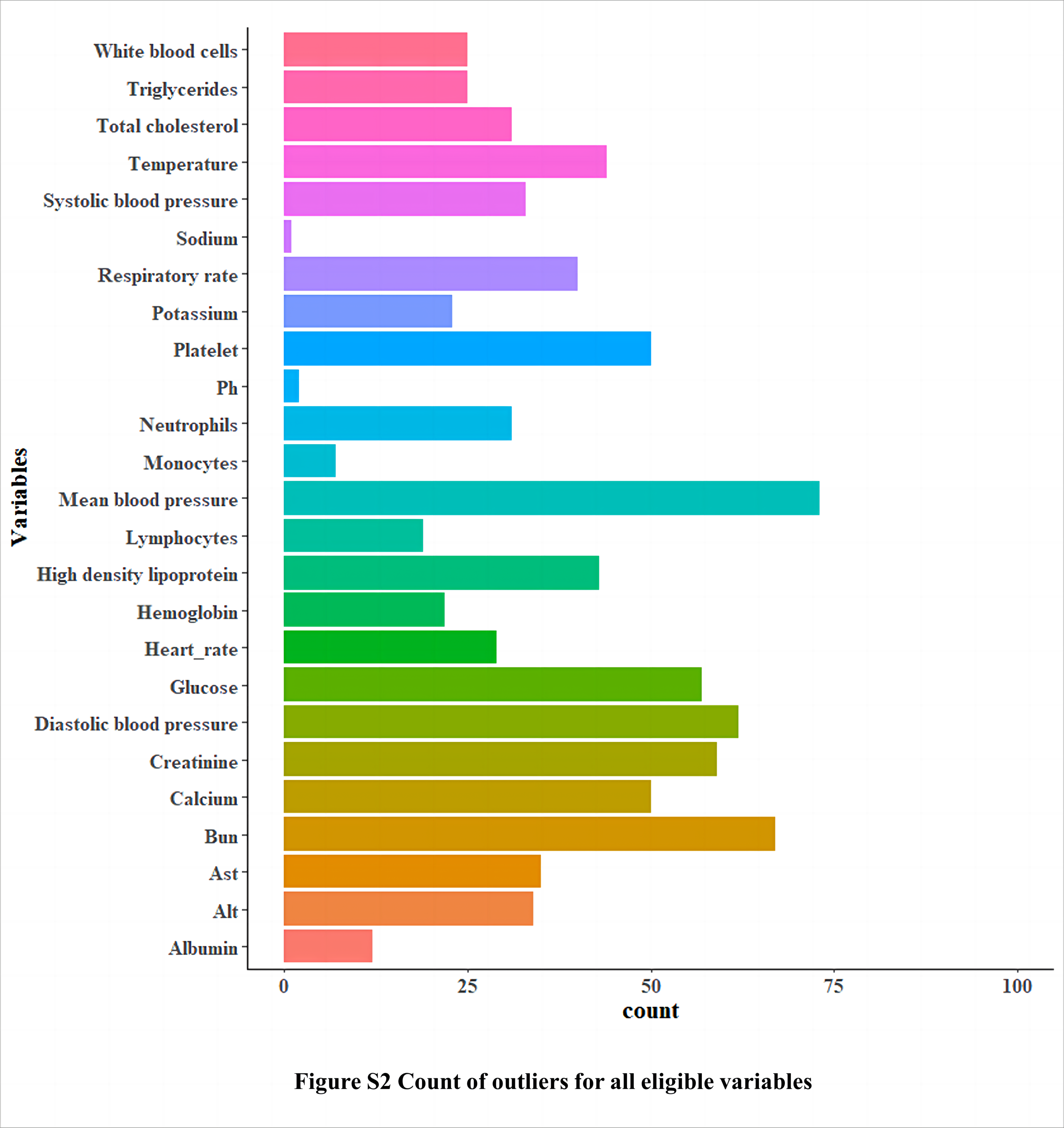

Supplement: Supplementary file 2 — Additional file 2: Figure S2. Count of outliers for all eligible variables [file 40001_2024_1756_MOESM2_ESM.png]

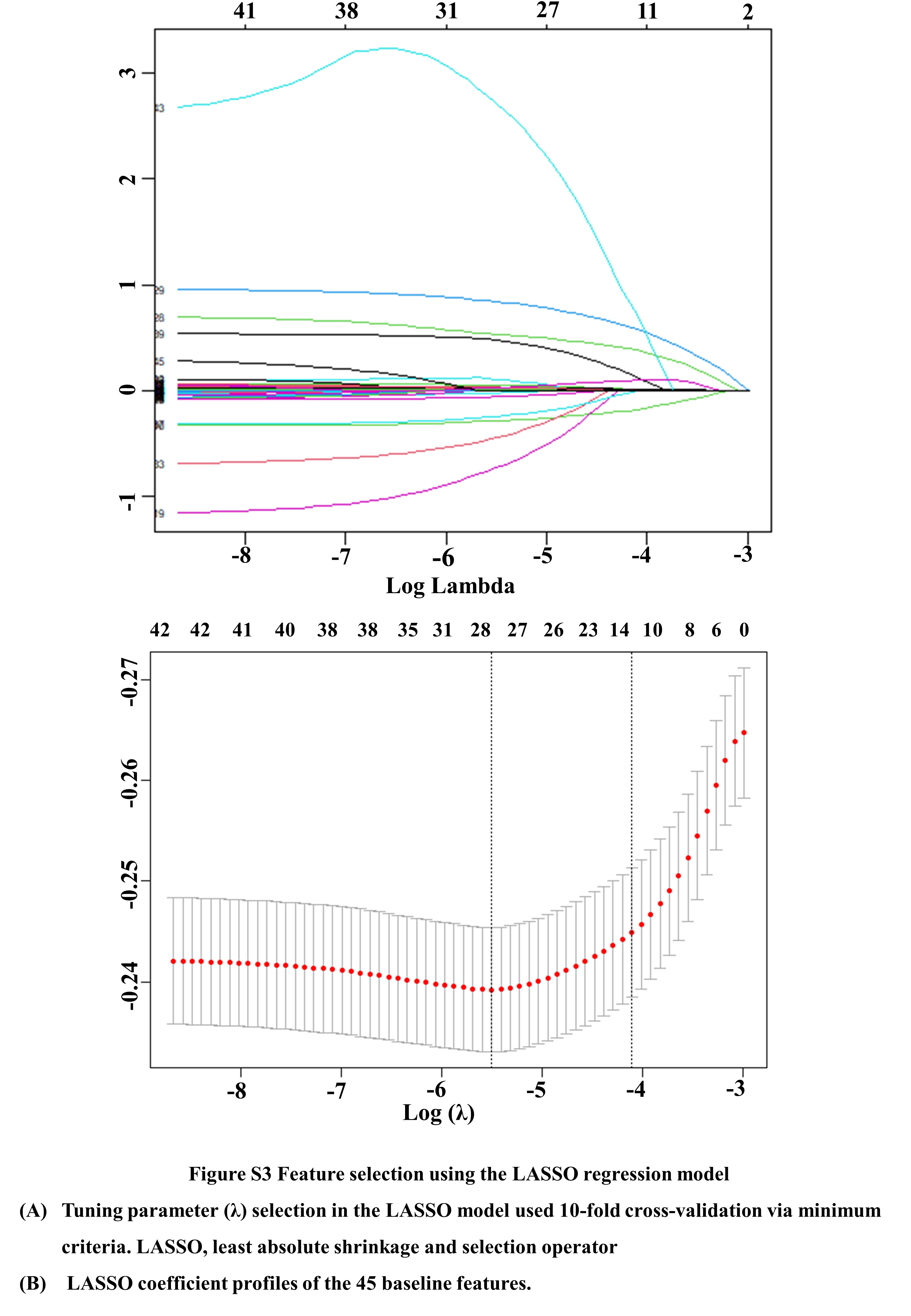

Supplement: Supplementary file 3 — Additional file 3: Figure S3. Feature selection using the LASSO regression model. (A) Tuning parameter (λ) selection in the LASSO model used tenfold cross-validation via minimum criteria. LASSO, least absolute shrinkage and selection operator. (B) LASSO coefficient profiles of the 45 baseline features. [file 40001_2024_1756_MOESM3_ESM.tif]

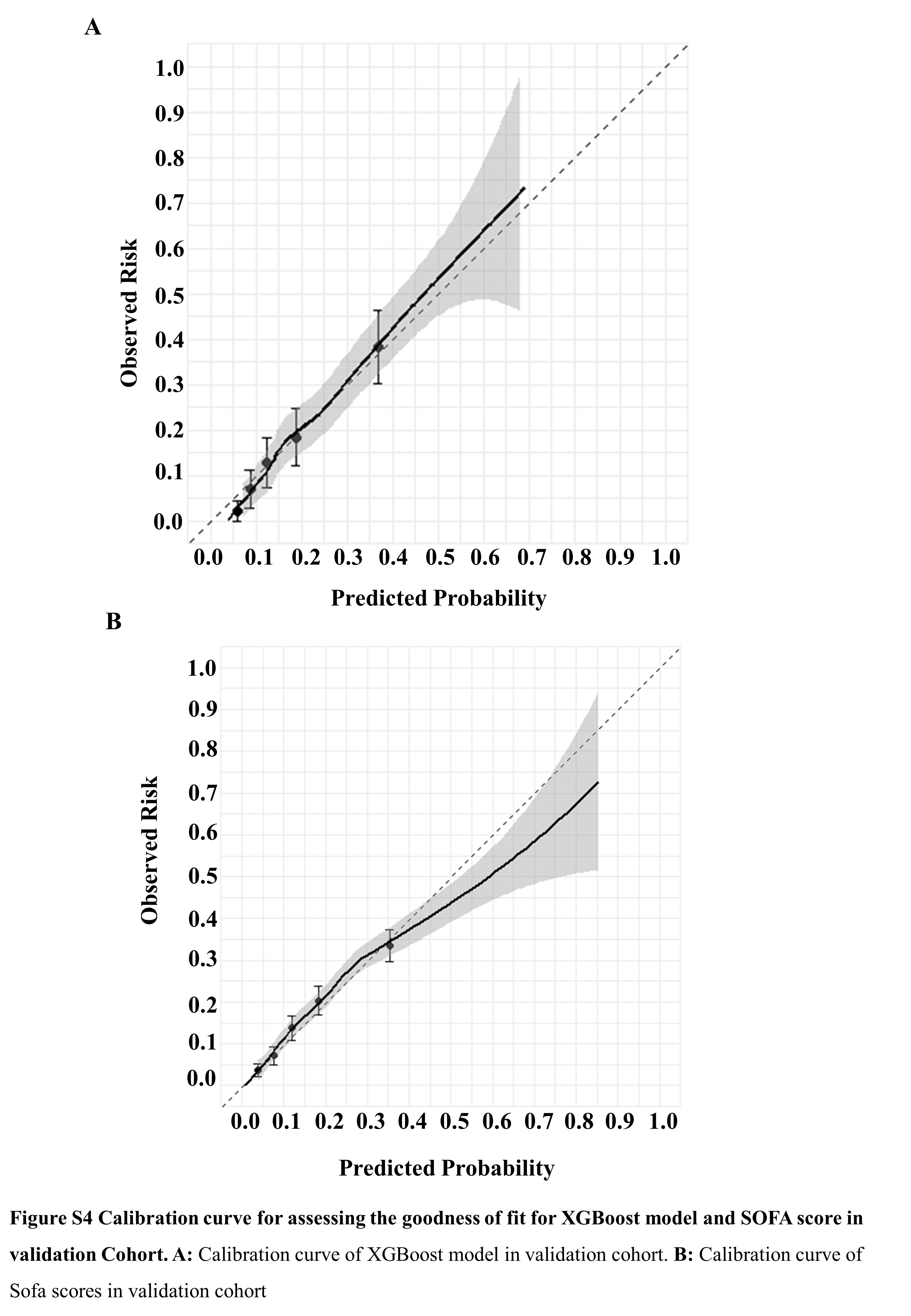

Supplement: Supplementary file 4 — Additional file 4: Figure S4. Calibration curve for assessing the goodness of fit for XGBoost model and SOFA score in validation Cohort. A: Calibration curve of XGBoost model in validation cohort. B: Calibration curve of Sofa scores in validation cohort. [file 40001_2024_1756_MOESM4_ESM.tif]

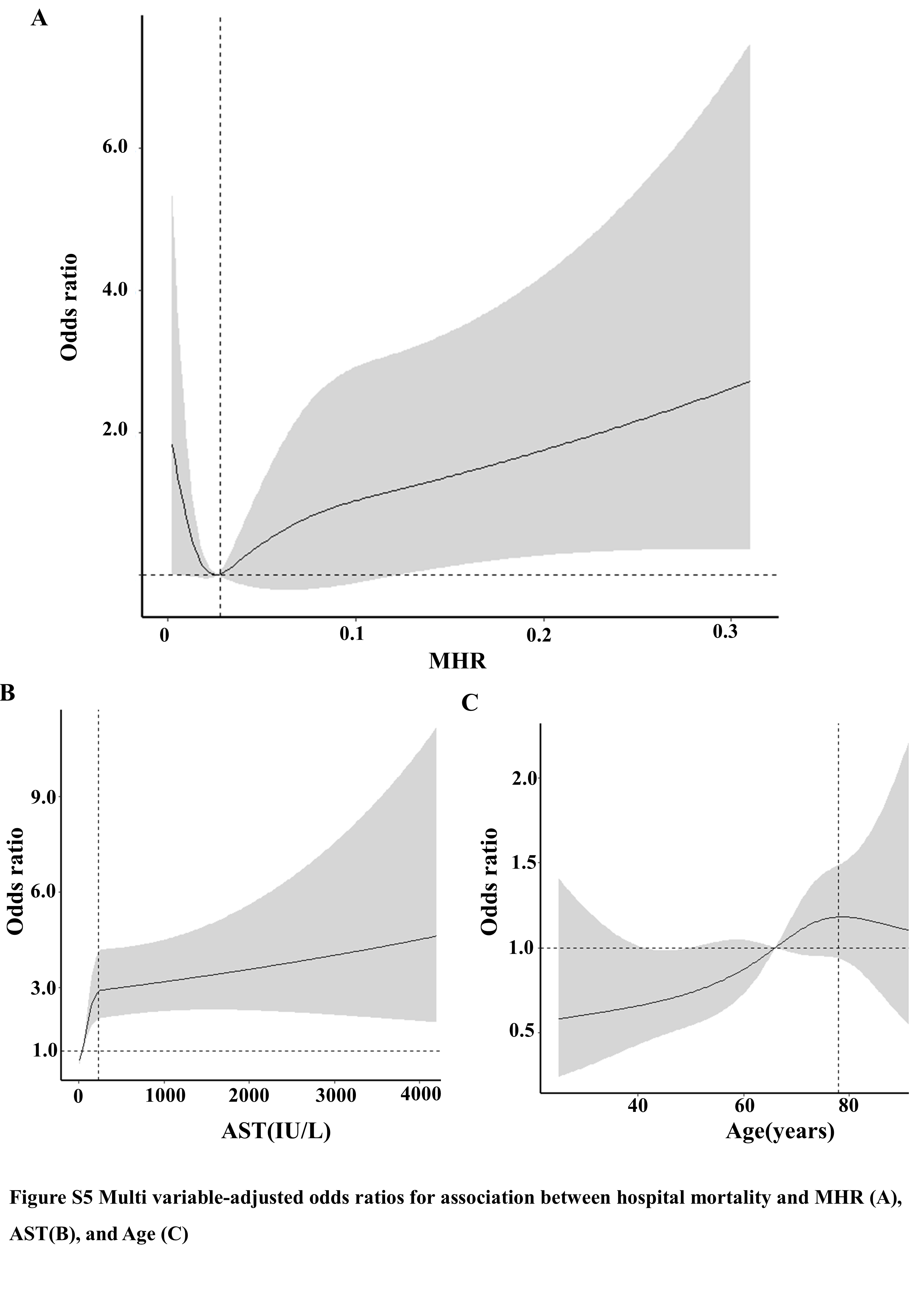

Supplement: Supplementary file 5 — Additional file 5: Figure S5. Multi variable-adjusted odds ratios for association between hospital mortality and MHR (A), AST (B), and Age (C). [file 40001_2024_1756_MOESM5_ESM.tif]
