# Supplementary material for: Predicting sepsis in-hospital mortality with machine learning: a multi-center study using clinical and inflammatory biomarkers
Source: Eur J Med Res. 2024 Mar 6;29:156. doi: 10.1186/s40001-024-01756-0 (PMC10918942; doi:10.1186/s40001-024-01756-0)
Supplement: Supplementary file 6 — Additional file 6: Table S1. Performances of the seven machine learning models and Sofa score for predicting in-hospital mortality from test cohort. [file 40001_2024_1756_MOESM6_ESM.docx]

**TableS1 Performances of the seven machine learning models and Sofa score for predicting in-hospital mortality from test cohort**

| **Model** | **AUC** | **Accuracy** | **Precision** | **Recall** | **F1 Score** |
| --- | --- | --- | --- | --- | --- |
| **XGBoost** | 0.771 | 0.846 | 0.872 | 0.95 | 0.96 |
| **Sofa score** | 0.702 | 0.844 | 0.854 | 0.91 | 0.94 |
| **Logistic regression** | 0.703 | 0.836 | 0.838 | 0.884 | 0.90 |
| **Random forest** | 0.677 | 0.832 | 0.845 | 0.853 | 0.903 |
| **K-nearest Neighbor** | 0.617 | 0.786 | 0.793 | 0.818 | 0.877 |
| **Naïve Bayes** | 0.690 | 0.828 | 0.834 | 0.867 | 0.909 |
| **SVM** | 0.658 | 0.830 | 0.825 | 0.858 | 0.896 |
| **Decision Tree** | 0.60 | 0.790 | 0.83 | 0.867 | 0.888 |

**XGBoost: extreme Gradient Boosting, SVM: Support Vector Machine, AUC: the area under curve**
