# Supplementary material for: Predicting sepsis in-hospital mortality with machine learning: a multi-center study using clinical and inflammatory biomarkers
Source: Eur J Med Res. 2024 Mar 6;29:156. doi: 10.1186/s40001-024-01756-0 (PMC10918942; doi:10.1186/s40001-024-01756-0)
Supplement: Supplementary file 7 — Additional file 7: Table S2. The Pearson correlation between variables. [file 40001_2024_1756_MOESM7_ESM.docx]

**Table S2 The Pearson correlation between variables**

|  | **Albumin** | **BUN** | **MHR** | **Potassium** | **AST** | **Age** | **Heart rate** | **NLR** | **NHR** |
| --- | --- | --- | --- | --- | --- | --- | --- | --- | --- |
| **Albumin** | 1.0 | -0.11 | -0.16 | -0.02 | 0.003 | 0.01 | -0.12 | -0.13 | -0.24 |
| **BUN** | -0.11 | 1.0 | 0.21 | 0.38 | 0.09 | 0.29 | -0.06 | 0.12 | 0.22 |
| **MHR** | -0.16 | 0.21 | 1.0 | 0.1 | 0.06 | -0.06 | 0.07 | 0.03 | 0.73 |
| **Potassium** | -0.02 | 0.37 | 0.1 | 1.0 | 0.17 | 0.04 | 0.007 | 0.007 | 0.12 |
| **AST** | 0.002 | 0.09 | 0.06 | 0.17 | 1.0 | -0.08 | 0.1 | 0.06 | 0.10 |
| **Age** | 0.02 | 0.29 | -0.06 | 0.044 | -0.08 | 1.0 | -0.12 | 0.07 | -0.07 |
| **Heart rate** | -0.12 | -0.06 | 0.07 | 0.007 | 0.1 | -0.12 | 1.0 | 0.12 | 0.13 |
| **NLR** | -0.13 | 0.12 | 0.03 | 0.007 | 0.06 | 0.07 | 0.12 | 1.0 | 0.39 |
| **NHR** | -0.24 | 0.22 | 0.73 | 0.12 | 0.10 | -0.07 | 0.13 | 0.4 | 1.0 |

**BUN:** Blood Urea Nitrogen; **AST:**Aspartate Aminotransferase; **MHR:** monocyte/high-density lipoprotein cholesterol ratio; **NHR:**the ratio of neutrophils to HDL; **NLR:**the neutrophil-to-lymphocyte ratio
