# Supplementary material for: Predicting sepsis in-hospital mortality with machine learning: a multi-center study using clinical and inflammatory biomarkers
Source: Eur J Med Res. 2024 Mar 6;29:156. doi: 10.1186/s40001-024-01756-0 (PMC10918942; doi:10.1186/s40001-024-01756-0)
Supplement: Supplementary file 8 — Additional file 8: Table S3. The distance correlation between variables. [file 40001_2024_1756_MOESM8_ESM.docx]

**Table S3 The distance correlation between variables**

|  | **Age** | **Albumin** | **AST** | **Bun** | **Heart Rate** | **MHR** | **NHR** | **NLR** | **Potassium** |
| --- | --- | --- | --- | --- | --- | --- | --- | --- | --- |
| **Age** | 1.0 | 0.06 | 0.10 | 0.25 | 0.137 | 0.09 | 0.079 | 0.089 | 0.0797 |
| **Albumin** | 0.062 | 1.0 | 0.05 | 0.14 | 0.124 | 0.184 | 0.25 | 0.125 | 0.05 |
| **AST** | 0.10 | 0.05 | 1.0 | 0.12 | 0.06 | 0.05 | 0.08 | 0.07 | 0.21 |
| **BUN** | 0.25 | 0.14 | 0.12 | 1.0 | 0.07 | 0.22 | 0.24 | 0.10 | 0.38 |
| **Heart Rate** | 0.13 | 0.12 | 0.06 | 0.07 | 1.0 | 0.09 | 0.12 | 0.13 | 0.05 |
| **MHR** | 0.09 | 0.18 | 0.05 | 0.22 | 0.09 | 1.0 | 0.77 | 0.06 | 0.10 |
| **NHR** | 0.07 | 0.25 | 0.08 | 0.24 | 0.12 | 0.77 | 1.0 | 0.32 | 0.10 |
| **NLR** | 0.08 | 0.12 | 0.07 | 0.1 | 0.13 | 0.06 | 0.32 | 1.0 | 0.04 |
| **Potassium** | 0.07 | 0.05 | 0.21 | 0.38 | 0.05 | 0.1 | 0.1 | 0.04 | 1.0 |

**BUN:** Blood Urea Nitrogen; **AST:**Aspartate Aminotransferase; **MHR:** monocyte/high-density lipoprotein cholesterol ratio; **NHR:**the ratio of neutrophils to HDL; **NLR:**the neutrophil-to-lymphocyte ratio
