# Supplementary material for: Predicting sepsis in-hospital mortality with machine learning: a multi-center study using clinical and inflammatory biomarkers
Source: Eur J Med Res. 2024 Mar 6;29:156. doi: 10.1186/s40001-024-01756-0 (PMC10918942; doi:10.1186/s40001-024-01756-0)
Supplement: Supplementary file 9 — Additional file 9: Table S4. The mutual information between variables. [file 40001_2024_1756_MOESM9_ESM.docx]

**Table S4 The mutual information between variables**

|  | **Age** | **Albumin** | **AST** | **BUN** | **Heart rate** | **MHR** | **NHR** | **NLR** | **Potassium** |
| --- | --- | --- | --- | --- | --- | --- | --- | --- | --- |
| **Age** | 2.3 | 0.07 | 0.04 | 0.1 | 0.05 | 0.04 | 0.04 | 0.06 | 0.04 |
| **Albumin** | 0.07 | 2.3 | 0.04 | 0.06 | 0.03 | 0.05 | 0.07 | 0.06 | 0.03 |
| **AST** | 0.04 | 0.04 | 2.3 | 0.05 | 0.04 | 0.04 | 0.04 | 0.04 | 0.05 |
| **BUN** | 0.1 | 0.06 | 0.05 | 2.3 | 0.05 | 0.05 | 0.06 | 0.04 | 0.11 |
| **Heart rate** | 0.05 | 0.03 | 0.04 | 0.05 | 2.3 | 0.04 | 0.04 | 0.06 | 0.03 |
| **MHR** | 0.04 | 0.05 | 0.04 | 0.05 | 0.04 | 2.3 | 0.44 | 0.04 | 0.04 |
| **NHR** | 0.05 | 0.07 | 0.04 | 0.06 | 0.04 | 0.44 | 2.3 | 0.12 | 0.04 |
| **NLR** | 0.06 | 0.05 | 0.04 | 0.04 | 0.06 | 0.04 | 0.12 | 2.30 | 0.03 |
| **Potassium** | 0.04 | 0.03 | 0.05 | 0.11 | 0.03 | 0.04 | 0.04 | 0.03 | 2.3 |

**BUN:** Blood Urea Nitrogen; **AST:**Aspartate Aminotransferase; **MHR:** monocyte/high-density lipoprotein cholesterol ratio; **NHR:**the ratio of neutrophils to HDL; **NLR:**the neutrophil-to-lymphocyte ratio
