# Supplementary material for: Predicting sepsis in-hospital mortality with machine learning: a multi-center study using clinical and inflammatory biomarkers
Source: Eur J Med Res. 2024 Mar 6;29:156. doi: 10.1186/s40001-024-01756-0 (PMC10918942; doi:10.1186/s40001-024-01756-0)
Supplement: Supplementary file 10 — Additional file 10: Table S5. The maximal information coefficient between variables. [file 40001_2024_1756_MOESM10_ESM.docx]

**Table S5 The maximal information coefficient between variables**

|  | Age | Albumin | AST | BUN | Heart Rate | MHR | NHR | NLR | Potassium |
| --- | --- | --- | --- | --- | --- | --- | --- | --- | --- |
| Age | 0.97 | 0.42 | 0.97 | 0.42 | 0.97 | 0.32 | 0.97 | 0.32 | 0.42 |
| Albumin | 0.42 | 0.98 | 0.98 | 0.42 | 0.33 | 0.33 | 0.33 | 0.42 | 0.33 |
| AST | 0.97 | 0.97 | 0.97 | 0.42 | 0.42 | 0.32 | 0.42 | 0.42 | 0.32 |
| BUN | 0.42 | 0.42 | 0.42 | 0.97 | 0.97 | 0.97 | 0.97 | 0.32 | 0.32 |
| Heart Rate | 0.97 | 0.32 | 0.42 | 0.97 | 0.97 | 0.42 | 0.97 | 0.42 | 0.42 |
| MHR | 0.32 | 0.32 | 0.32 | 0.97 | 0.42 | 0.97 | 0.42 | 0.42 | 0.32 |
| NHR | 0.97 | 0.32 | 0.42 | 0.97 | 0.97 | 0.42 | 0.97 | 0.42 | 0.42 |
| NLR | 0.33 | 0.42 | 0.42 | 0.32 | 0.42 | 0.42 | 0.42 | 0.97 | 0.97 |
| Potassium | 0.42 | 0.32 | 0.32 | 0.32 | 0.42 | 0.32 | 0.42 | 0.97 | 0.97 |

**BUN:** Blood Urea Nitrogen; **AST:**Aspartate Aminotransferase; **MHR:** monocyte/high-density lipoprotein cholesterol ratio; **NHR:**the ratio of neutrophils to HDL; **NLR:**the neutrophil-to-lymphocyte ratio
